# Predicting the mutational drivers of future SARS-CoV-2 variants of concern

**DOI:** 10.1126/scitranslmed.abk3445

**Published:** 2022-01-11

**Authors:** M. Cyrus Maher, Istvan Bartha, Steven Weaver, Julia di Iulio, Elena Ferri, Leah Soriaga, Florian A. Lempp, Brian L. Hie, Bryan Bryson, Bonnie Berger, David L. Robertson, Gyorgy Snell, Davide Corti, Herbert W. Virgin, Sergei L. Kosakovsky Pond, Amalio Telenti

**Affiliations:** ^1^ Vir Biotechnology, San Francisco, California 94158, USA; ^2^ Department of Biology Institute for Genomics and Evolutionary Medicine Temple University, Philadelphia, PA 19122; ^3^ Computer Science and Artificial Intelligence Laboratory, Massachusetts Institute of Technology, Cambridge, MA 02139, USA.; ^4^ Ragon Institute of MGH, MIT, and Harvard, Cambridge, MA 02139, USA.; ^5^ Department of Biological Engineering, Massachusetts Institute of Technology, Cambridge, MA 02139, USA.; ^6^ Department of Mathematics, Massachusetts Institute of Technology, Cambridge, MA 02139, USA.; ^7^ Computer Science and Artificial Intelligence Laboratory. Massachusetts Institute of Technology, Cambridge, MA 02139, USA.; ^8^ MRC-University of Glasgow Centre for Virus Research, University of Glasgow, Glasgow GS1 1QH, UK; ^9^ Department of Pathology and Immunology, Washington University School of Medicine, St. Louis, MO 63110, USA; ^10^ Department of Internal Medicine, UT Southwestern Medical Center, Dallas, TX 75390, USA

## Abstract

SARS-CoV-2 evolution threatens vaccine- and natural infection-derived immunity as well as the efficacy of therapeutic antibodies. To improve public health preparedness, we sought to predict which existing amino acid mutations in SARS-CoV-2 might contribute to future variants of concern. We tested the predictive value of features comprising epidemiology, evolution, immunology, and neural network-based protein sequence modeling, and identified primary biological drivers of SARS-CoV-2 intra-pandemic evolution. We found evidence that ACE2-mediated transmissibility and resistance to population-level host immunity has waxed and waned as a primary driver of SARS-CoV-2 evolution over time. We retroactively identified with high accuracy (area under the receiver operator characteristic curve, AUROC=0.92-0.97) mutations that will spread, at up to four months in advance, across different phases of the pandemic. The behavior of the model was consistent with a plausible causal structure wherein epidemiological covariates combine the effects of diverse and shifting drivers of viral fitness. We applied our model to forecast mutations that will spread in the future and characterize how these mutations affect the binding of therapeutic antibodies. These findings demonstrate that it is possible to forecast the driver mutations that could appear in emerging SARS-CoV-2 variants of concern. We validate this result against Omicron, showing elevated predictive scores for its component mutations prior to emergence, and rapid score increase across daily forecasts during emergence. This modeling approach may be applied to any rapidly evolving pathogens with sufficiently dense genomic surveillance data, such as influenza, and unknown future pandemic viruses.

## Teaser

Simple epidemiologic metrics can accurately predict which mutations in the SARS-CoV-2 genome will increase in frequency over the coming months.

## INTRODUCTION

SARS-CoV-2 evolution presents an ongoing challenge to public health. Tens of thousands of mutations have arisen in the SARS-CoV-2 genome as the pandemic has progressed. Understanding the relative importance of mutations in viral proteins, particularly those of relevance for antiviral immunity, is key to allocating preparedness efforts. Mutations in the viral Spike protein have received particular attention because Spike is the target of antibody-mediated immunity and is the primary antigen in current vaccines (*1*). As of December 1st, 2021, there are 10,381 distinct amino acid substitutions, insertions, or deletions in Spike sequences from the GISAID database (*2*). These mutations occur at all but one position in the protein, in different combinations, creating over 160,000 unique Spike protein sequences. A small subset of these mutations are components of “Variants Being Monitored” (VBMs), “Variants of Interest” (VOIs) or “Variants of Concern” (VOCs), as classified by the United States Centers for Disease Control (CDC) (*3*). The distinction between VOIs and the higher alert VOCs is whether a negative clinical impact is suspected or confirmed. VBMs are variants that would be classified as VOCs if not for low prevalence.

Early statistical and algorithmic identification of the key Spike amino acid changes contributing to future putative VBM/VOI/VOCs are of clear benefit to public health strategy. Such predictions could enhance the identification of vulnerabilities for antibody-based therapeutics, vaccines, and diagnostics. Predicting future successful mutations would extend the time available to develop proactive responses at earlier stages of spread. It would also complement existing forecasting efforts which seek to predict overall SARS-CoV-2 incidence, hospitalizations, and death over time (*4*–*6*). Focus on the success of individual mutations rather than genomic variants also facilitates longer-term forecasting. The combinatorics of modeling genomic variants quickly become intractable. As a toy example, for a protein of length 1200, there are over 250 million distinct sequences that differ by only two amino acid changes. By focusing on amino acid success from the outset, we rely on common and largely correct assumptions about independence between mutations, and are able to leverage more information per mutation, thus extending the timeline on which evolution can be meaningfully forecast.

There is a robust and expanding set of analyses characterizing the features of amino acid mutations of SARS-CoV-2. Studies have identified the emergence of new variants with altered biological or antigenic properties (*7*–*9*) and characterized them using low-throughput methods (*10*, *11*). Deep mutational scanning elucidates the in vitro biological effects of all single site amino acid substitutions in a fixed genomic backbone (*12*–*14*). Others have characterized the distribution of immunodominant sites across the viral proteome (*15*, *16*) and estimated the fitness of viral sequences using neural natural language processing (NLP) applied to protein sequences (*17*).

We sought here to build upon these data and approaches to forecast the mutations that will spread from season to season. We hypothesized that this would also allow us to identify the dominant biological drivers of viral evolution over short-term timescales. These two goals are mutually reinforcing: the features that are most useful for forecasting can be inferred as measuring viral fitness. Conversely, a better understanding of evolutionary dynamics can make modeling more accurate and robust. To accomplish these goals we described patterns of rapid mutation spread both globally and within the United States and elucidated the relative predictive importance of amino acid mutational features comprising immunity, transmissibility, evolution, language model, and epidemiology. Next, we utilized data from previous infection waves to train and back-test a forecasting model that anticipates future spreading mutations and illustrated how forecasted mutations could differentially affect clinical antibodies. We extended this analysis to forecast mutations, specifically on the Delta lineage, across the whole SARS-CoV-2 proteome. As the number of Omicron sequences increases, such a targeted analysis could be repeated for that lineage as well.

## RESULTS

### Biological and epidemiological features of SARS-CoV-2 mutations that spread

**Fig. 1.**
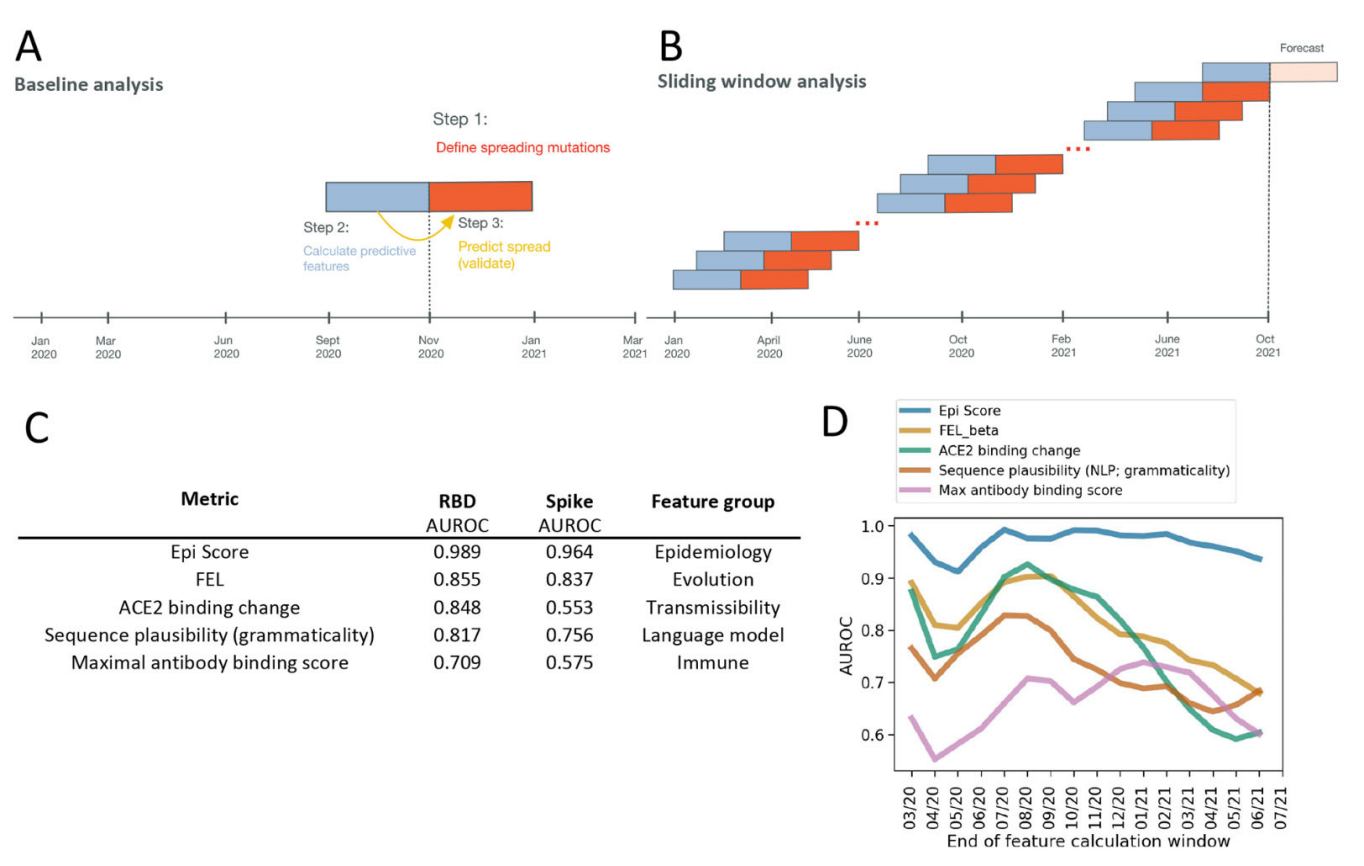
**Predicting mutation spread. (A)** Analyzing performance at baseline and over time. The core analysis consists of three steps. First, creating a working definition for spreading mutations. Second, calculating features that can predict future spread using a window of prior data. Third, having constructed models on training data, run prediction of future spread (Forecast), and interpret the results. **(B)** Performance was assessed over time by repeating this analysis in sliding time windows covering the whole data collection period. **(C)** The most predictive metrics within each feature group at baseline (see **Table 1** and **table S1**) were ranked by performance within the receptor binding domain (RBD), where the most data are available and for the Spike. **(D)** RBD classification accuracy over time for the top GISAID-based feature (Epi Score), and the top transmission and immune variables (**Table 1**). AUROCs in panel D are smoothed with a rolling window of two analysis periods. AUROC, area under the receiver operating characteristic curve. FEL, fixed effects model for detecting site-wise selective pressure.

For the purpose of developing the models, we defined “spreading” amino acid mutations as a specified fold change in frequency across multiple countries, comparing time windows before and after a chosen date (**Fig. 1**). These mutations could be substitutions, insertions, or deletions. (*2*) Within each country, we tabulated the number of sequences containing the mutation being modeled, versus those that did not, in the three months before and after a date of interest (**Fig. 1A**). For each mutation, we calculated a fold change and an associated comparison-adjusted p-value. Mutations with a significant Benjamini-Hochbert adjusted p-value (q < 0.05) from any country were retained. This set was further filtered using the following empirical criteria, all of which had to be met to define a mutation as spreading: a fold change (FC) from baseline of at least 10.0 in at least one country; a FC of at least 2.0 across three or more countries; and a minimum global frequency of 0.1% in the later time window. We highlight that the sequences used to calculate fold change from baseline and minimum frequency were all collected after those used for model training or feature calculation, with no overlap or interleaving between the two datasets. Performance was assessed over time by repeating this analysis in shifting or sliding time windows covering the whole data collection period, which corresponded to the three months prior to the desired forecast start date (**Fig. 1B**). Assessed data windows ranged from January-March 2020 to June-August 2021.

This definition of spreading mutations captured the expansion of VOI/VOCs globally (**fig. S1A**) as well as the growth of a number of lesser-known mutations (**fig. S1B**). Implicit in a mutation-centric approach to forecasting is the assumption that mutations accumulate in a manner that is approximately independent, or at least that their interactions can be averaged out when looking across all genomic backgrounds. To test for significant violations of this implicit assumption, we tested for linkage between all pairs of spreading mutations (**fig. S2**). Enrichment for co-occurrence between pairs of mutations at a rate of greater than 8-fold was observed for fewer than 5% of mutation pairs. Thus, we find that (pairwise) independence between mutations is a useful and approximately correct simplifying assumption.

We next determined which features of amino acid mutations are informative for predicting their spread at baseline (**Table 1****, data file S1**). Within the receptor binding domain (RBD) of Spike, we found that ACE2 binding affinity was a useful predictor of mutation spread (area under the receiver operator characteristic curve, AUROC=0.85; **Fig. 1C**). Another useful predictor was the change in in vitro expression of Spike mutants (AUROC=0.82; **fig. S3A**). Among measures of immune escape, the binding contributions of known antibody epitopes (antibody binding score) to anti-SARS-CoV-2 antibodies were predictive of mutation spread (AUROC=0.71; **Fig. 1C**) whereas CD4^+^ or CD8^+^ T-cell immunogenicity did not offer substantial explanatory power for mutation spread (AUROC=0.52-0.62; **fig. S3A**). We found that Natural Language Processing (NLP) scores for sequence plausibility (grammaticality) (*17*) were similarly predictive to deep mutational scanning data (AUROC=0.82; **Fig. 1C**). The best evolutionary feature for prediction of spread (AUROC=0.86; **Fig. 1C**) was obtained from Fixed Effects Likelihood (FEL (*18*)) from the Hyphy package [http://www.hyphy.org] (*19*) which tests for pervasive negative or positive selection across the internal branches of a phylogenetic tree.

**Table 1.** Summary of analytical features. A total of 48 parameters for 14 variables were created for 5 feature groups. These features capture evolutionary, immune, epidemiologic, transmissibility, and language model predictors of mutation spread. A detailed description of all parameters is included in **data file S1**.

**Feature group**	**Variable**	**Meaning**	**Source or reference**	**Number of parameters**
Evolution	Positive selection (FEL, MEME)	Parameters from Fixed Effects Likelihood (FEL) and Mixed Effects Model of Evolution (MEME)	HyPhy (*19*)	11
	Codon-SHAPE	RNA SHAPE constraint	Manfredonia *et al*. 2020 (*32*)	3
	Viral entropy	Shannon entropy at each codon position for an amino acid site	This work	3
Immune	CD8 epitope escape	The frequency of SARS CoV-2 mutations in cytotoxic lymphocyte (CTL) epitopes	Agerer *et al*. 2021 (*15*)	1
	CD8 response	The percent and average CD8+ T cell response to an epitope in patients	Tarke *et al*. 2021 (*33*)	2
	CD4 response	The percent and average CD4+ T cell response to an epitope in patients	Tarke *et al*. 2021 (*33*)	2
	Antibody binding score	The estimated percent contribution of a site to binding of the indicated antibody, as estimated by Molecular Operating Environment (MOE)	This work	17
	Maximum escape fraction in vitro	The maximum escape fraction across all conditions for that mutation	Greaney *et al*. 2021 (*34*)	1
Epidemiology	Variant frequency	The percent of sequences with the mutation	Calculated from GISAID (*2*)	1
	Fraction of unique haplotypes	The fraction of unique Spike haplotypes in which a mutation is observed	Calculated from GISAID (*2*)	1
	Number of countries	The number of countries where it has been observed.	Calculated from GISAID (*2*)	1
	Epi Score	The exponentially weighted mean rank across the other epidemiology variables	Calculated from GISAID (*2*)	1
Transmissibility	RBD expression change	Change in RBD expression due to the mutation	Starr *et al*. 2020 (*13*)	1
	ACE2 binding change	The change in binding affinity for ACE2	Starr *et al*. 2020 (*13*)	1
Language model	Language model	Grammaticality and semantic change of a mutation	Hie *et al*. 2021 (*17*)	2

The highest predictive performance, however, was obtained from epidemiological features, that is, variables which more directly measure sampled mutation counts (**Table 1**). The most predictive variable in this feature category was “Epi Score”, the exponentially weighted mean ranking across the other epidemiological variables (mutation frequency, fraction of unique haplotypes in which the mutation occurs, and the number of countries in which it occurs), with AUROC=0.99. This score captures both lineage expansion and recurrent mutation that occurs in multiple variant lineages by convergent evolution. We note that the utility of recurrent mutation signals is consistent with recent findings that convergent evolution plays a substantial role in SARS-CoV-2 adaptation (*20*). As observed for the RBD alone, within Spike we also obtained the best predictive performance with epidemiologic (AUROC=0.96) and evolutionary (AUROC=0.84) measures (**Fig. 1C**). The performance of other feature sets for spike is presented in **fig. S3B**.

We next sought to interrogate the robustness of this approach to changes in the underlying drivers of SARS-CoV-2 evolution. For example, it has been hypothesized that selection due to immune pressure has increased with time as more individuals became immune through infection or vaccination (*20*). For example, the Gamma P.1 lineage is thought to have spread rapidly in Brazil largely due to immune selection in a population with high seroprevalence (*21*). We measured the predictive performance of antibody binding scores, which quantify the predicted percent contribution of each Spike site to antibody affinity. We took this metric as a proxy for B cell immunodominance (**Table 1**) (*22*). Taking the maximum of this value across antibodies at a given site yielded the maximum antibody binding score. The predictiveness of this metric increased from nearly uninformative early in the pandemic (p-value for difference from random=0.53), to an AUROC of 0.75 (p<1e-4; **fig. S2C**) for predicting spreading mutations during the third wave of the pandemic (**Fig. 1D**). Predictiveness subsequently decreased again to 0.64 by summer of 2021 coincidental with the emergence of Delta. However, we found that epidemiological features maintained their performance, achieving an AUROCs of 0.92-0.97 over multiple evaluation periods (**Fig. 1D**).

Last, we trained models to predict spreading mutations using all, or various subsets of, the features identified above. We employed logistic regression with baseline features as inputs. The best predictors were epidemiologic features (AUROC=0.98) and positive selection features (AUROC=0.83; **fig. S4A**). The performance of the full model was comparable to the non-model-based performance of Epi Score (**fig. S4B**). Therefore, to simplify reproducibility and further minimize the risk of overfitting, we used Epi Score to predict mutation spread going forward. We found that taking the top 5% of mutations according to their Epi Score achieved reasonable sensitivity (~50%) and maintained a positive predictive value of between 20 and 60% across time windows (**fig. S5**). Given that an average of ~3% of observed mutations are spreading at any point in time, this represents more than a 300-fold improvement in sensitivity, and a 6- to 20-fold improvement in positive predictive value relative to random selection.

In summary, immunity, transmissibility, evolution, language model, and epidemiologic features all effectively predicted mutation spread. The methodology captured changes to the underlying selective forces over the course of the pandemic. We found that epidemiologic features in particular display superior accuracy and maintain it over time.

### Examining global dynamics and the emergence of VOCs

To determine whether local or global dynamics drive mutation spread, we examined whether spreading mutations in the United States were better predicted by global or US-only epidemiological values. We tested the performance of Epi Score across four waves of the pandemic. We found that mutations were predicted with an AUROC above 0.85 up to 11 months in advance, both within the United States and globally. Global epidemiology metrics were best overall and were generally more predictive of country-level mutation spread than the country-level metrics themselves (**fig. S6**).

**Fig. 2.**
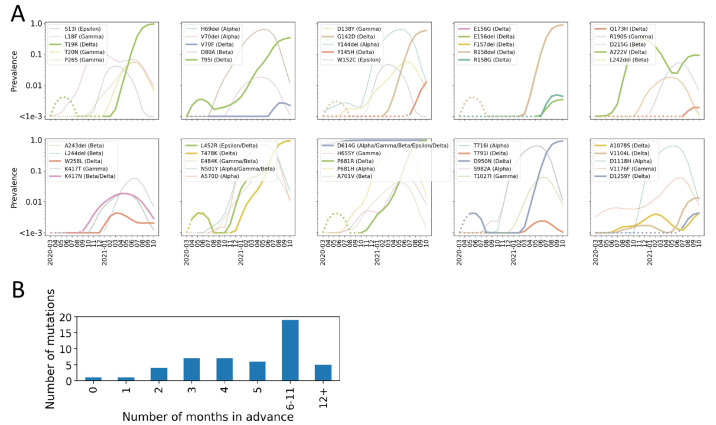
**Early detection of variant mutations. (A)** Depiction of where in their growth trajectories current and former VOC/VOI mutations were first forecast to spread. Dotted lines denote the part of the curve where the variant had not yet been forecast to spread. Solid lines denote the period after first forecast. Delta-defining variants are shown by thick lines. Mutations are presented in genomic order. (**B**) The number of months between when the mutations presented in (A) were forecast and when they reached a prevalence of 1% globally.

To illustrate the practical utility of Epi Score using global features, we assessed how early we would have been able to forecast the spread of Spike mutations that define current and former CDC VOCs, VOIs, and VBMs (n=50 defining mutations). To be conservative, we defined the date that a mutation was first forecast as the earliest date at which it was predicted to spread in two subsequent analysis periods. Of the 50 mutations (**Fig. 2A**), the median time between when a mutation was forecast to spread and when it reached 1% frequency was 5 months. The maximum was 20 months, while the minimum was 0 months for D614G, because this mutation had already reached a frequency of 69% by the first forecast period. The distribution of these forecast intervals is presented in **Fig. 2B**.

Of particular note, Y145H was forecast to spread starting in July of 2021. This mutation is now a defining mutation of AY.4.2, a spreading sub-lineage of the Delta VOC. As of October 2021, AY.4.2 accounted for 8.5-11.3% of samples in the UK. Estimated growth rates remain slightly higher for AY.4.2 than for Delta, and the household secondary attack rate was higher for AY.4.2 cases than for other Delta cases (*23*). Based on these observations, we conclude that our approach was able to predict key mutations, across all current and former VOC/VOI/VBMs, several months in advance. Early warning of mutations in current VOCs, VOIs, and VBMs would have been possible before reaching worrisome degrees of global spread.

### Understanding performance through a causal lens

**Fig. 3.**
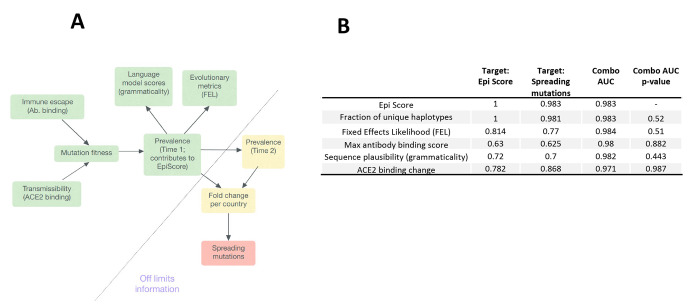
**Epi Score mediates effects captured by other data sources. (A)** Causal model: mutation fitness drives viral prevalence at time 1 (as measured by global frequency, and geographic and haplotype distribution, Epi Score). Language model score or evolutionary metrics are summaries of GISAID data and therefore are shaped by mutation prevalence. Prevalence at time 1 predicts prevalence at time 2, which ultimately leads to mutation being defined as spreading. Therefore, prevalence at time 1 (as captured by Epi Score) mediate the effects of the biological variables that enhance viral fitness through transmissibility or escape adaptation. **(B)** To quantitatively test for mediation, we assessed whether variables were better at predicting mutations in the top 5% of Epi Scores, compared to spreading mutations for time 2 versus time 1. “Combo AUC” refers to the combined AUC of that variable with Epi Score. Significant improvements of the combined model over that of Epi Score alone would indicate complementarity, and therefore predictive information not captured by Epi Score alone.

Seeking to understand the high predictive performance of epidemiologic features, we constructed a directed acyclic graph to represent the hypothesized causal relationships, and to probe whether relative trends in performance were consistent with the expectations that follow from this model (**Fig. 3A**). We proposed that epidemiologic features mediate the relationship between viral fitness and mutation spread. Our rationale was that if a mutation’s contribution to viral fitness was sufficient to drive it to appreciable prevalence at one time point (as measured by global frequency and geographic distribution), and in the context many genetic backgrounds, it would likely drive it to higher prevalence in the future as well (unless it were outcompeted by a more fit adaptation, or the fitness landscape changed). This type of mediated relationship (fitnessÞcurrent prevalenceÞfuture prevalence) implies that epidemiological prevalence features will capture information from both known and unknown drivers of selection.

If the causal model were reasonable, we would expect first that variables whose causal effects are mediated, as defined above, should predict epidemiologic variables at a comparable or even greater accuracy compared to spreading mutations. This is illustrated by comparing the first and second columns of **Fig. 3B**. We observed that, with the exception of the maximal antibody binding score, all top variables predicted Epi Scores better than they predict mutation spread. The lower predictiveness of maximal antibody binding score for Epi Scores would be consistent with a slight time lag effect due to shifting evolutionary pressures.

A second criterion for mediation is that information from these variables should not substantially complement the predictiveness of the epidemiologic variables alone. In other words, there should be little or no additional information that other inputs provide relative to the epidemiologic variables. We assessed this by comparing the AUROCs of two-variable models in column 3 of **Fig. 3B** with the AUROC for Epi Score alone (0.983). The only nominal AUROC increase for a complemented model was observed for the evolutionary measure FEL (0.984). We did not find statistically significant complementarity with Epi Score for this or any other variable, either within the RBD or across full length Spike (see supplemental section “Mediation Analysis”, **table S1).**

Our examination of mediated causal relationships begins by assuming a causal graph based on prior knowledge. Such an approach is common to many causal inference methods (*24*) and represents a well-understood limitation of these methods (*24*). Therefore, we considered this as a tool to more systematically analyze the plausibility of our results. Although it is generally difficult to verify the structure of proposed causal graphs, our findings support the concept that epidemiological variables mediate the effects of other classes of explanatory variables, and this may explain their high predictive accuracy.

### Emergence and spread of Omicron

**Fig. 4.**
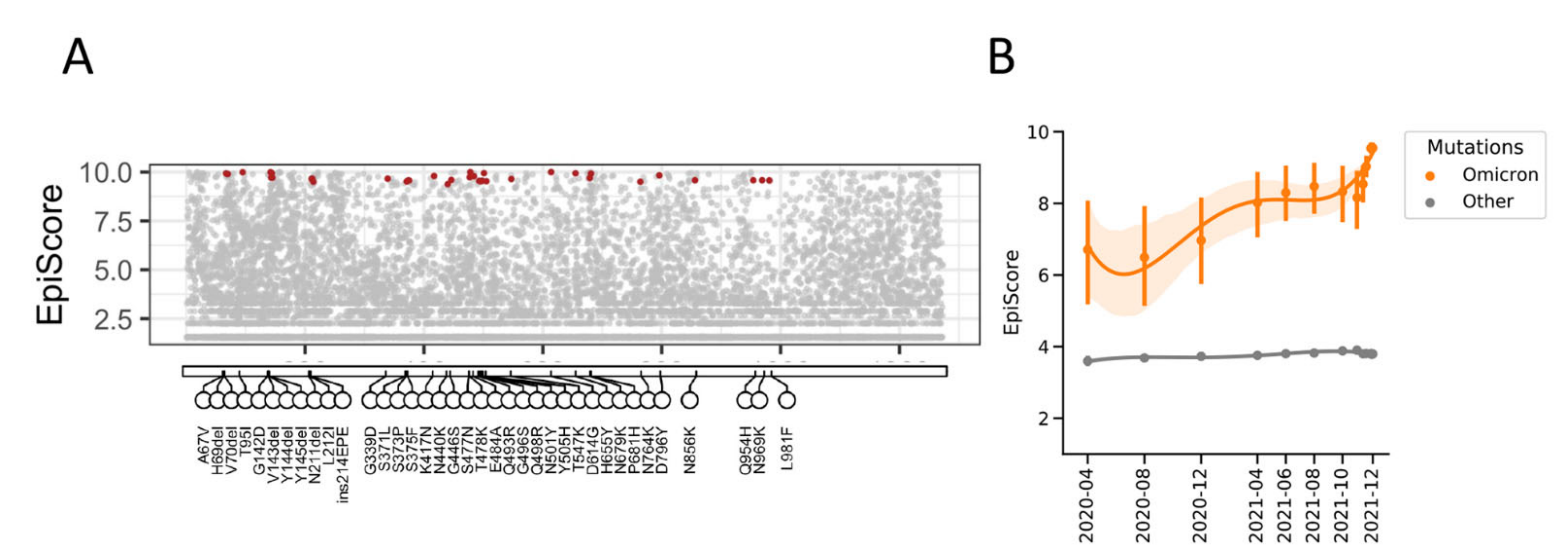
**Emergence and spread of Omicron. (A)** The Epi Scores of 37 Omicron-defining mutations are shown as of December 8, 2021 (red dots). **(B)** Although some of the mutations in Omicron already had very high Epi Scores and were widely spread, emergent mutations were distinguished by the progressively increasing Epi Score between April 2020 and August 2021 preceding the rapid acceleration at the end of 2021. Shown are mean and confidence interval Epi Score values. Other: Epi Score of all other mutations in the SARS-CoV-2 spike.

While this work was in revision, we were confronted with the emergence in late November 2021 of the Omicron (B.1.1.529/21K) variant. Despite the low frequency of many of the individual mutations that define the major haplotype of Omicron (median allele frequency 0.00046), we observed high Epi Score values across Spike (median Epi Score of 9.51); **Fig. 4A**. A benefit of the computational simplicity of Epi Score is that predictions can easily be updated on a daily basis. We therefore sought to move beyond single time point Epi Scores to examine trends in Epi Score across time for the Omicron mutations. The time-analysis showed that the Omicron Spike mutations had progressively higher Epi Score values long preceding the acceleration that characterized the emergence of Omicron in November 2021 (**Fig. 4B**). We additionally found that the spread of Omicron was rapidly reflected in the raising Epi Scores of its mutations, and that daily forecasts allowed the identification of trending scores.

As an independent approach to assess the singularity of Omicron, we also examined the evolutionary nature of the Omicron mutations using our language model. Omicron had a grammaticality change between that of Alpha and Delta, but the highest semantic change (predicted antigenic shift) of any SARS-CoV-2 lineage (**fig. S7**). Indeed, Omicron’s semantic change score was twice that of both Alpha and Delta, consistent with high levels of mutation and immune escape adaptation.

### Forecasting spreading mutations in Spike and proteome-wide

Building upon the accurate prediction of spreading mutations across different waves of the pandemic, we next leveraged Epi Score on current data to forecast mutations that may contribute to VOIs and VOCs over the coming months. Because global metrics outperformed metrics restricted to the United States, even for forecasting within the United States, we focused on global forecasting. We considered shortening our feature calculation window to further mitigate the effects of shifting evolutionary dynamics. However, we found that longer feature calculation windows improved performance across all prediction windows (**fig. S8**).

**Fig. 5.**
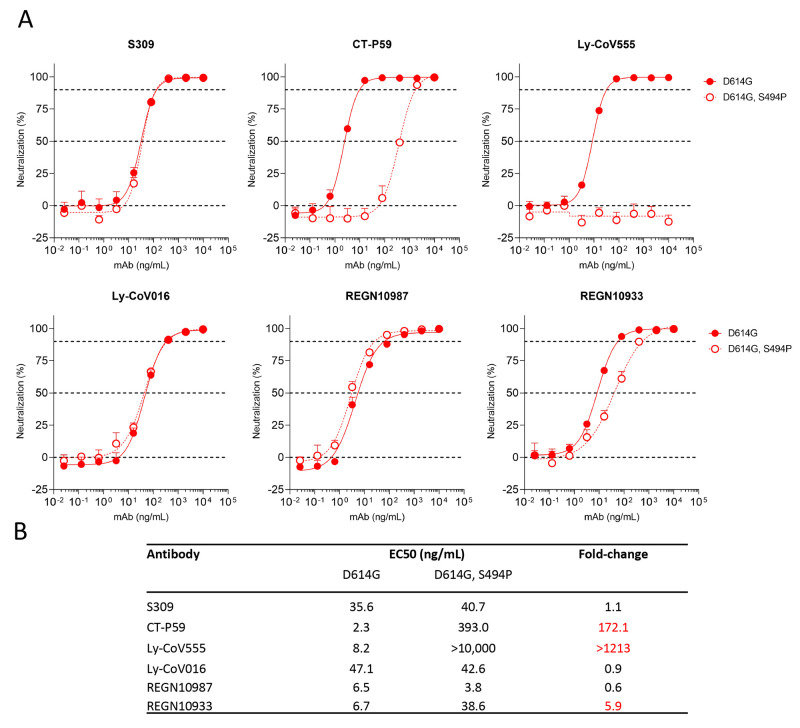
**S494P mutation decreases neutralization potential of three clinically approved therapeutic antibodies. (A)** VSV-SARS-CoV-2 pseudovirus was generated based on the “Wuhan-Hu-1” sequence with either the D614G mutation or D614G and S494P mutations. Virus neutralization was measured in a microneutralization assay on Vero E6 cells. Example results from one repeat are shown. **(B)** EC50 values and fold-changes were calculated from two independent experiments. S309 is the parent molecule of VIR-7831, which had been previously evaluated on the S494P variant and showed no change in neutralization (*25*)·

As an application of the forecasting analysis, we examined how forecasted mutations intersected with the binding sites of clinical antibodies as of October 19^th^, 2021. We found wide variation in the number of forecasted mutations per antibody epitope (**Table 2**), ranging from 10 mutations for Celltrion’s CT-P59, to two low-frequency mutations for Vir-7831 (sotrovimab), which was designed to be more robust to viral evolution by targeting a region that is conserved across coronaviruses (*25*). The two mutations in the epitope of sotrovimab, A340S and R346K, do not limit neutralization (*25*, *26*). As an additional proof of concept, we focused our attention on Spike S494P, a mutation reported to have enhanced binding affinity to ACE2 (*27*), and to reduce neutralization by 3-5-fold in some convalescent sera (*27*). We found that the S494P mutation decreases neutralization potential of clinical therapeutic antibodies: Ly-CoV555 (bamlanivimab), CT-P59 and to a lesser extent to REGN10933 (casirivimab) (**Fig. 5****)**.

**Table 2.** Forecasted mutations for therapeutic antibodies. Forecasted mutations, as of October 19th (including VOC mutations) were intersected with the binding epitopes of therapeutic monoclonal antibodies. Mutations were included if they were in sites contributing at least 1% of the total binding energy for a given antibody, as estimated by Molecular Operating Environment (MOE) program. Mutations known to decrease antibody EC50 more than five-fold are marked with asterisks. Mutations with daggers indicate neutralization is decreased less than five-fold (https://covdb.stanford.edu/page/susceptibility-data/), whereas values with double daggers indicate untested antibody, mutation combinations.

**Clinical therapeutic antibody**	**Forecasted mutations in epitopes**
VIR-7831 (sotrovimab)	A344S†, R346K†
LY-CoV016 (etesevimab)	K417T‡, K417N*, L455F‡
REGN10987 (imdevimab)	R346K†, K444N*, G446V*
LY-CoV555 (bamlanivimab)	L452R*, L452Q‡, V483F†, E484K*, E484Q*, F490S*, S494L‡, S494P*
REGN10933(casirivimab)	K417T*, K417N*, L455F*, G476S*, S477I‡, T478K‡, E484K*, E484Q*, F490S*
CT-P59	K417T‡, K417N†, L452R*, L452Q‡, L455F‡, E484K*, E484Q‡, F490S‡, S494L‡, S494P‡

**Fig. 6.**
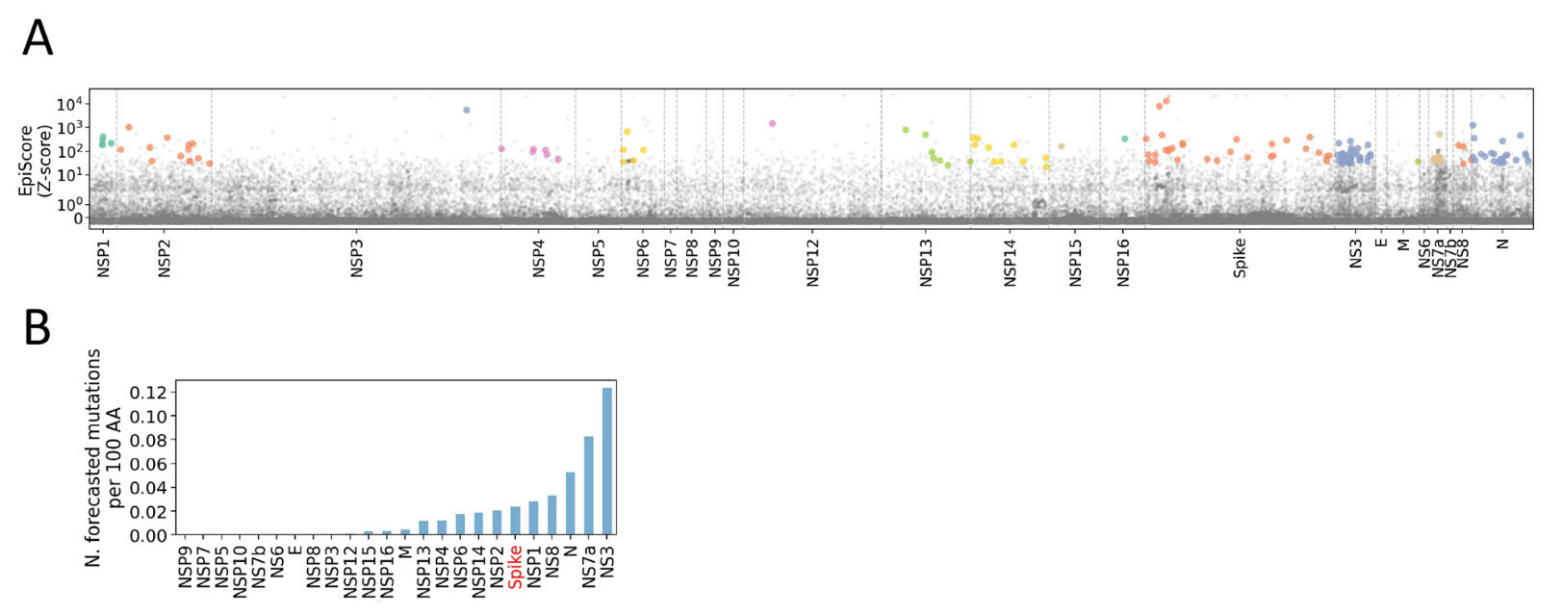
**Manhattan-style plot of Epi Scores across the SARS-CoV-2 Delta proteome. (A)** For visualization purposes, Epi Scores have been calculated as Z-scores, which correlate to the default, rank-based calculation as a spearman R > 0.99. Points highlighted in color occur at a frequency over 0.1% on a Delta background (B.1.617.2 + AY lineages) and occur at significantly positively selected sites (FEL FDR-adjusted q-value < 0.05). All mutations occurring at over 80% frequency, in the lineages accounting for >80% of all Delta cases, were excluded from the visualization. Thus, the plot serves to highlight variants predicted to spread and under positive selection in the current Delta background. For a complete listing, S**uppl. File S2. (B)** The rate per 100 amino acids of highlighted forecasted mutations from panel A, per gene in the SARS-CoV-2 proteome.

Last, to demonstrate the flexibility and extensibility of our approach, we forecasted the spread of mutations specifically on the Delta genomic background, across the full SARS-CoV-2 proteome. Because the components of Epi Score can be calculated for any mutation where sequencing data are available, extension to the full proteome is trivial and not computationally taxing. It can also be reasonably calculated on any subset of sequences to determine which mutations are most likely to spread based on their characteristics within that subset (or lineage). Therefore, it is also straightforward to adapt this approach to produce lineage-specific forecasts. **Fig. 6A** shows a Manhattan-style plot of Epi Scores across the full SARS-CoV-2 genome. The plot highlights all mutations at positively selected sites (FEL, fixed effects model for detecting site-wise selective pressure, FDR < 0.05) that currently occur at a frequency over 0.1% on a Delta background. We found 151 such mutations, distributed across the proteome. The mutation density was 1.8 per 100 amino acids across the whole proteome, with a rate varying from 0 to 12.3 across SARS-CoV-2 proteins (**Fig. 6B**). By this measure, the highest mutational density was identified in ORF3/NS3, an accessory protein that is reported to modulate autophagosome–lysosome fusion (ORF3a) (*28*) and antagonize interferon (Orf3b) (*29*). Spike was close to average, with a density of 2.3 mutations per 100 amino acids. Based on the Epi Score ranking, the top 5 mutations for potential to spread were Spike:G142D, Spike:T95I, NSP3:A1711V, N:Q9L, and NSP2:K81N. All mutation Epi Scores proteome-wide are presented in **data file S2**.

In summary, we established a method for predicting spreading mutations and applied it to forecast future contributors to putative VOCs/VOIs/VBMs. These predictions yield mutations known to be important from in vitro data. We conclude that this approach can anticipate spreading mutations many months in advance. We find that a subset of forecast mutations could have implications for the continued efficacy of clinical antibodies, but that the level of these effects varies widely. We then extended our analysis to encompass the full SARS-CoV-2 proteome, and to produce Delta and informative Omicron forecasts. This work also suggests that there is considerable potential for spreading mutations located outside of Spike, underlining the importance of forecasting methods that can be applied across the whole viral proteome.

## DISCUSSION

We established a working definition for spreading mutations and leveraged this definition to deliver a systematic analysis of amino acid features predictive of mutation spread. This yielded a simple, explainable, and accurate approach for forecasting mutations several months in advance, across multiple pandemic waves. Calculating this scoring was also efficient enough to enable daily forecast updates on millions of sequences using only a laptop. Although this strategy required nothing more than genomic surveillance data, we also highlighted the value of the complete mapping of epitopes, in vitro deep site-directed mutagenesis, and downstream functional experimental validation. Confidence in the prediction of spreading mutations came through retrospectively evaluating multiple waves of the pandemic and verifying consistency with experimental data, and with a plausible causal framework. Furthermore, long observed lags between the earliest warning signals and high population frequency of current mutations in VOCs, VOIs, and VBMs gave further support for using forecasting to anticipate the spread of future concerning mutations. Although this approach will be limited in its ability to anticipate mutations that appear and rise to high frequencies within a short time frame, we found this to be a rare occurrence.

We evaluated epidemiologic features aggregated in the Epi Score such as mutation frequency, and the distribution of mutations across countries and fraction of unique haplotypes across which a mutation occurs. We explored other predictors, including the rate of increase of each of these features, but did not find that they improved performance. We note that the fraction of unique haplotypes shared similarities to phylogenetic measures of recurrent mutation. However, there is considerable lack of phylogenetic resolution in such calculations, so the number of recurrent mutations is a statistically “noisy” measure, depends strongly on the method used to build phylogenies, and is very expensive to compute. The fraction of unique haplotypes, on the other hand, is fast to compute, can be perfectly estimated, and will increase with both recurrent mutation and single-lineage expansion; both of which are indicative of a positive contribution to fitness.

Omicron emerged a*s* the paper was completing the review process. Despite the limited numbers of viral sequences available as of December 2021, we observed a distinctive pattern of Omicron mutations that, despite low frequency of many individual mutations, already had high Epi Score values. It is also notable that for all mutations, high Epi Score values antedated the emergence of Omicron, even though those mutations had not yet converged on the same haplotypes. We interpret these data as indicative that individual mutations were endowed with advantageous properties in the viral genome even before their co-occurrence on the Omicron spike.

There are limits to this study; general prediction of viral evolution is fundamentally an intractable problem. The current work only addresses a simpler question: predicting which mutations will increase in frequency over some threshold in the near future based on the analysis of their recent patterns of spread. Thus, the study predicts spread of existing mutations, but not a true emergence of previously unobserved mutations. In addition, it is difficult to predict which lineages, i.e., a major viral haplotype, will spread because this would require the complex projection of growth of multiple mutations together. These limitations notwithstanding, the data on Omicron suggest that successful lineages may be defined by the convergence of mutations that, individually, exhibited high Epi Score values and other features that signal adaptive evolution.

Although this work forecasts which mutations will spread, the success of a given mutation does not necessarily result in clinical or public health consequences. Therefore, we posit that the value of the predictions is to prioritize mutations for functional screening. Here, we demonstrate how a subset of spreading mutations differentially impact clinical antibodies. We also extended the analysis to encompass the whole viral proteome. By this approach, we identified spreading amino acid replacements in other viral proteins, and highlighted positions under strong positive selection. Given the limited understanding of the role of non-Spike regions of the proteome in driving the pandemic, we believe that those non-Spike mutations should be prioritized for understanding their role in evading innate immunity, increasing the replication of SARS-CoV-2, and more generally for their contribution to viral fitness. We intend for these results to provide a foundation for future improvement. Although we have shown that Epi Score is robust to shifting evolutionary dynamics, performance can be monitored in real-time, and if necessary, re-tuned to capture novel behavior as now shown with the emergence of Omicron. This approach can also be generalized and improved upon to stay ahead of evolutionary cycles for other pathogens (*30*), when sufficiently rich and representative genomic sampling is available.

## MATERIALS AND METHODS

**Study Design.** *Sample size.* The current work to define spreading amino acid mutations was based on viral sequences and metadata obtained from GISAID EpiCoV project (https://www.gisaid.org/). A total of 4,487,305 sequences were analyzed.

*Research objectives.* We hypothesized that the pattern of spread could be estimated from the large database of GISAID. Next, we hypothesized that one or more variable comprising biological, immunological, epidemiological and genomic (including language) features could be identified as drivers of the spread.

*Experimental design.* We used predictive models and expressed predictive performance using the area under the receiver operator characteristic curve (AUROC). Prediction was performed using forward feature selection followed by logistic regression. The criterion for forward selection was cross-validated AUROC of the logistic regression model within the training set. Feature selection and model fitting were performed separately within each fold of the outer cross validation loop. Logistic regression was chosen due to its sample efficiency.

**Statistical analysis.** Spreading mutations were defined based on a Fisher’s exact test for frequency fold change per country, adjusted for multiple comparisons, followed by filters for rate of spread (max fold change of at least 10, fold change > 2 in three or more countries), and a minimum prevalence of 0.1%. We estimated epistasis using pointwise mutual information, which corresponds to the log ratio of the observed prevalence of a pair to the expected prevalence assuming independence. The most predictive variable, “Epi Score” was defined as the exponentially weighted mean ranking across the other epidemiological variables (mutation frequency, fraction of unique haplotypes in which the mutation occurs, and the number of countries in which it occurs. For natural language processing (NLP) neural network features, we used the grammaticality and semantic change scores reported by Hie *et al*. (*17*) in which a bidirectional long short-term memory (BiLSTM) model was trained on Spike sequences from GISAID and GenBank. Natural selection features were generated using MEME (*31*) and FEL (*18*) methods implemented in the HyPhy package (*19*) (version 2.5.31). Mediation analysis was based on the Baron and Kenny test. The list of forecast mutations was generated by calculating Epi Scores on the most recent three months of data and taking the top 5% of mutations, a cutoff chosen based empirical analyses.

## Supplementary Materials

### This PDF file includes:

Figs. S1 to S8 Table S1 References (*35*–*41*)

### Other Supplementary Material for this manuscript includes the following:

Data files S1 to S3

MDAR Reproducibility Checklist
