# Supplementary material for: Predicting the mutational drivers of future SARS-CoV-2 variants of concern
Source: Sci Transl Med. 2022 Jan 11:eabk3445. doi: 10.1126/scitranslmed.abk3445 (PMC8939770; doi:10.1126/scitranslmed.abk3445)
Supplement: Supplementary file 1 — Figs. S1 to S8 Table S1 References (35–41) [file scitranslmed.abk3445_sm.pdf]

Science Translational Medicine
AAAS

Supplementary Materials for

Predicting the mutational drivers of future SARS-CoV-2 variants of concern

M. Cyrus Maher *et al.*

Corresponding authors: M. Cyrus Maher, cmaher@vir.bio; Amalio Telenti, atelenti@vir.bio

DOI: 10.1126/scitranslmed.abk3445

The PDF file includes:

Materials and Methods
Figs. S1 to S8
Table S1
References (*35–41*)

Other Supplementary Material for this manuscript includes the following:

Data files S1 to S3
MDAR Reproducibility Checklist

Supplemental materials and methods, figures and table

Predicting the mutational drivers of future SARS-CoV-2 variants of concern
M. Cyrus Maher[1]*, Istvan Bartha[1], Steven Weaver[2], Julia di Iulio[1], Elena Ferri[1], Leah Soriaga[1], Florian A. Lempp[1], Brian L. Hie[3,4], Bryan Bryson[4,5], Bonnie Berger[6,7], David L. Robertson[8], Gyorgy Snell[1], Davide Corti[1], Herbert W. Virgin[1], Sergei L. Kosakovsky Pond[2], Amalio Telenti[1]*

Materials and Methods

Variable definitions and data sources. The definitions of the variables presented, how they are grouped into categories, and where they can be retrieved, can be found in **data file S1**.

Code environment. Analyses on GISAID data extracts were conducted in python (Python Software Foundation. Python Language Reference, version 3.7. Available at http://www.python.org). Code was built in Jupyter lab notebooks*(35)* and relied upon a number of common data analysis libraries*(36–39)*.

Sequence access. Viral sequences and metadata were obtained from GISAID EpiCoV project (https://www.gisaid.org/). Analysis was performed on sequences submitted to GISAID up to October 19th, 2021. SARS-CoV-2 protein sequences were obtained directly from the protein metadata file provided by GISAID. A total of 4,487,305 sequences were analyzed.

Defining spreading mutations. As described in the main text, mutations were selected based on a Fisher's exact test for frequency fold change per country, adjusted for multiple comparisons. This approach was selected after exploratory analysis found that more granular trend tests such as the Mann-Kendall trend test were less favorably powered in low-data countries. Multiple comparisons were adjusted for using the function *statsmodels.stats.multitest.fdrcorrection* from the *statsmodels* package*(38)* with an alpha of 0.05. This applies a Benjamini-Hochberg correction. We constructed the 2x2 tables used for the Fisher's exact test in the following manner. Within each country, we tabulated four counts: the number of sequences containing the mutation of interest, versus those that did not; in one window before, and one after the date cutoff (Nov. 1st 2020). From each table, we calculated a fold change and an associated comparison-adjusted p-value. Mutations with a comparison-adjusted p-value less than 0.05 from any country were accepted. The number of comparisons for the adjustment was taken as the number of countries times the number of observed mutations worldwide.

Epistasis calculation. We estimated epistasis using pointwise mutual information, which corresponds to the log ratio of the observed prevalence of a pair to the expected prevalence assuming independence. The expansion of a lineage will introduce a positive correlation between mutations, violating this assumption in a way that makes the result conservative in estimating the level of epistasis. That is, the expected co-occurrence rate will be underestimated, so the observed prevalence will look proportionally larger relative to expectation, increasing estimated levels of epistasis. To reduce the noise from small sample effects, we removed mutation pairs with an expected prevalence below 1/100,000. We also removed from consideration all mutation pairs that occurred at the same site.

Analyzed features. We investigated B cell epitopes and CD4+ and CD8+ T epitopes in the viral Spike protein*(15, 33)* as features that might predict spreading mutations. We also integrated *in vitro* mutagenesis data quantifying ACE2 binding of the viral Spike protein, expression of the viral spike protein, and escape from monoclonal antibody neutralization as measured in pseudovirus assays and/or binding of monoclonal antibodies to the Spike protein*(13, 34)*. In addition, we examined features of viral genome conservation such as RNA secondary structure constraint*(32)* and conservation of amino acids, as quantified by Shannon entropy, across the three sarbecovirus clades that encompass both SARS and SARS-CoV-2. We also assessed metrics of positive selection via MEME and FEL dN/dS-based methods*(40)*. We looked at variation in the viral proteome as captured by novel natural language learning tools*(17)*. We also evaluated epidemiologic features calculated from the training periods, such as mutation frequency, and the distribution of mutations across countries and viral variant backgrounds. We further calculated an integrated epidemiology score ("Epi Score") as the exponentially weighted mean ranking across mutation frequency, the fraction of unique variant sequences that contain an amino acid mutation, and the number of countries in which a mutation was been observed. Briefly, to calculate Epi score, we calculated the percentile of each component score (p), and from this calculated a new score (10^p). The average of these scores between the metric pair resulted in the combined score that ranged between 1 and 10.

Preparing feature sets. Deep mutational scan data*(34, 41)*, were retrieved from the following repository: https://github.com/brianhie/viral-mutation. For T cell data where scores are associated to oligonucleotides instead of mutations or sites, overlapping scores were averaged per site. When there were multiple experimental conditions, the maximum value per site was taken.

Antibody binding energies were calculated using Molecular modeling software MOE*(22)* (v2019.0102). To produce the antibody binding score, we first calculated pairwise binding energies (the sum of van der Waals, ionic, aromatic, and hydrogen-bond interactions) between each residue in the antigen epitope and each residue in the corresponding antibody Fab paratope, including all residues within a cutoff distance of 5.0 Å from the epitope/paratope interface. All structures were prepared prior to these calculations using the structure preparation, protonation and energy minimization steps in MOE, with default settings. The binding energies of each epitope residue that interacted with multiple Fab residues were added together and the percentage of the binding energy contributed by each epitope residue to the total binding energy was calculated. When more than one copy of the complex was present in the asymmetric unit, binding energy contributions were averaged across all copies. An overall binding energy per site was calculated as the max score across all antibodies.

Interspecies conservation was calculated from a nucleotide multiple sequence alignment of 44 sarbecoviruses. The Shannon entropy was calculated for each column in the alignment. Non-ATGC nucleotides (including gaps) were ignored. RNA structure SHAPE-seq intensities are downloaded from:
http://incarnatolab.com/downloads/datasets/SARS_Manfredonia_2020/XML.tar.gz)*(32)*. They were post processed by taking the mean of each 12-nucleotide sliding window and the window centered on a given nucleotide was used.

For natural language processing (NLP) neural network features, we used the grammaticality and semantic change scores *(17)* in which a bidirectional long short-term memory (BiLSTM) model was trained on Spike sequences from GISAID and GenBank. We obtained two versions of the model: the original model trained on sequences through sampled prior to June 1, 2020, and a second model that was retrained starting from random weight initializations on GISAID Spike sequences sampled prior to November 1, 2020. For all prediction periods after November 1st, the latter model was used. For prediction periods before this time, the former model was employed.

Natural selection features were generated using MEME*(31)* and FEL*(18)* methods implemented in the HyPhy package*(19)* (version 2.5.31). Data preparation, alignment, and tree inference were performed using an existing pipeline (https://github.com/veg/SARS-CoV-2/tree/compact/). Briefly, the pipeline curates sequences to remove low quality genomes and filter out potential sequencing errors and compresses the input to unique haplotypes over each gene region. A codon-aware mapping and multiple sequence alignment of gene regions is followed by rapid phylogenetic tree inference, and site-level selection analyses applied to internal tree branches (a standard procedure for viral intra-species data). FEL tests for pervasive negative or positive selection, while MEME tests for episodic positive selection. Both tests report p-values (based on the likelihood ratio tests); MEME further reports the number of branches which provide support for the positive selection model component. These data can be viewed at and downloaded from https://observablehq.com/@spond/selection-profile.

For epidemiologic variables, the "fraction of unique haplotypes" metric was defined as the proportion of the known haplotype backgrounds in which a given mutation occurred. The "mutation frequency" metric is defined as the fraction of sequenced individuals who had a mutation at that site. The "number of countries" metric is defined as the number of countries in which a mutation is observed in at least two sequences.

Epi Score was calculated as an exponentially weighted mean of the mutation ranks according to mutation frequency, fraction of unique haplotypes in which the mutation occurs, and the number of countries in which it occurs. Specifically, this involved (i) calculating the percentile for each score, for each metric, (ii) transforming the percentile via $p \rightarrow 10^p$ and (iii) averaging these exponentiated percentiles. The effect of this procedure is to assign highly differentiated weights to high rankings, and relatively small and similar weights to mutations that are not at the top of the list. For example, top ranked mutation versus a 90th percentile mutation will have a score difference of 2.1 (10 vs 7.9), whereas a mutation at the 50th percentile and one at the 40th percentile will have a score difference of 0.65 (3.16 vs 2.51). This scheme is particularly advantageous if measurements for lower-ranked entities are more noisy than higher ranked ones, or if one wants to up-weight high rankings.

For conversion from mutation- to site-level scores, the site level score was taken to be the maximum of mutation scores at that position. For the conversion of site- to mutation-level scores, the site-level score was assigned to all observed mutation at that position. In cases where data needed to be imputed, min-imputation was performed. For example, all sites without measured antibody binding energies were assigned a binding energy of zero. For all metrics, in cases of multiple experimental conditions, the max score per site or mutation (as appropriate) was taken. This was appropriate because the few metrics where lower scores implied a higher probability of spread (according to MEME p-values) did not have missing values.

Quantifying predictive performance. Predictive performance was quantified using the area under the receiver operator characteristic curve (AUROC). This quantity can be interpreted as the probability that a given score correctly ranks a random pair of positive and negative examples. Performance was assessed by two methods: (i) direct univariate ranking and (ii) model fitting with sets of features. The AUROC for univariate ranking was calculated as the maximum AUROC upon sorting by that metric in either ascending or descending order. Receiver Operator Characteristic (ROC) curves were generated by varying the numerical cutoff 'C' on each metric beyond which a mutation is called to be spreading. Given these calls, we then calculated sensitivity and specificity values for each value of C tested. Plotting sensitivity versus specificity yields the ROC curve. The area under the ROC (AUROC) was then used to quantify the capacity for that variable to distinguish spreading from non-spreading amino acid mutations.

For model fitting, performance was assessed by cross-validation. This involves partitioning the data into chunks or "folds" and iteratively predicting each (test) fold based on training with all the other chunks. In this procedure, it is important to make sure that correlated observations are kept in the same fold so that information does not leak between the training folds and the test fold. As a hypothetical example, a model could memorize the attributes of one identical twin in a training set to predict the values of the other in the test set. In the case of mutations, we were concerned that the co-occurrence of mutations on the same haplotypes could introduce a correlation in their metrics. To mitigate this issue, we made sure mutations from the same clade were always included in the same fold. Clades were defined according to GISAID annotation. The following clades were used to define folds: G, GH, GR, GRY. The remaining smaller clades were pooled into a single fold. This resulted in five folds ranging in size from around 700 to 2000 mutations. AUROC values were then calculated within each test fold and averaged across test folds to yield an overall performance.

Predictive performance of sets of features. Prediction was performed using forward feature selection followed by logistic regression. The criterion for forward selection was cross-validated AUROC of the logistic regression model within the training set. Feature selection and model fitting were performed separately within each fold of the outer cross-validation loop. Logistic regression was chosen due to its sample efficiency. We found that random forest classifiers obtained worse performance. Similarly, we found that combined models did worse than individual features if there was no feature selection step. We also tried a select K best feature selection strategy, which generally recapitulated the performance of the single best feature. We interpret these results to mean that limited sample size amplifies the effect of noisy features, and that greedily selecting for high AUROC features did not do a good job of selecting for complementarity. The members of each of the feature sets are enumerated in **data file S1.**

Selected features. Because a different model was fit for each cross-validation fold, we retrained a single model on all data to produce a single set of selected features for each feature set.

Mediation analysis. The strength of predictions based solely on the epidemiological features led us to consider a hypothesized causal model (**Fig. 4C**) to explain the effectiveness of these features relative to the contribution of biological measurements. We proposed that the biological factors that we analyzed determined viral fitness, in turn driving spread as measured via epidemiology. As illustrated in (**Fig. 4C**), epidemiology and evolution-based measures both draw on empirical variation, as captured by GISAID. We hypothesized that epidemiologic variables demonstrated superior performance because they are most proximal to the outcome variable, and therefore mediate the effects of the other variables. In causal inference, a mediated variable is a quantity that indirectly contributes to an outcome of interest (in this case spreading mutations) by altering an intermediate factor (a mediator; that is, initial mutation spread). The classical Baron and Kenny test for mediation can be divided into three steps: (i) make sure the variable of interest predicts the outcome, (ii) verify that the variable of interest predicts the mediator, and (iii) show that the variable of interest does not add to the predictive performance of the mediator when including both in a single model.

Step 1 was performed as part of the baseline analysis, and the complete results of this can be found in **figs. S2A and S2B**. For step 2, since few variables showed above-random performance outside of the RBD, we focused our analysis within the RBD. We attempted to predict the putative mediator (Epi Score). We predicted this surrogate outcome by first binarizing it to indicate whether the mutation score was in the top N mutations, where N is two times the number of mutations that spread in the observed dataset. We chose to multiply by two after consulting the positive predictive values in **fig. S5.** These results, shown by comparing the first and second columns in **Fig. 4C**, demonstrate that variables that are predictive of spread are also predictive of our epidemiologic predictor, with approximately the same magnitude. Therefore, we can conclude that criteria 1 & 2 were fulfilled.

Last, we fit models with each variable in addition to the epidemiologic predictor to test for complementarity. Because we were most interested in the RBD due to data availability, we encountered the issue that supervised models trained on full length spike tended to perform poorly with variables that are only observed within the RBD. Specifically, we saw that supervised models decreased in performance when including these variables, indicating overfitting. To address this issue and make our results more comparable to the univariate analysis, we generated a single score from the variable pairs by exponentially weighting the ranks of each metric. This was performed according to the same procedure as the Epi Score. Specifically, we calculated the percentile of each score (p), and from this calculated a new score (10 ** p). The average of these scores between the metric pair resulted in the combined score.

Testing integrated predictive models across waves and time lags. For testing predictive models across different waves and time lags, below are the time periods that we used for wave 2. The first group denotes the feature calculation window, and the second group of dates in each set denote the time window in which variant growth was assessed.

Below are the time periods used for wave 1
['2020-01', '2020-02', '2020-03'] ['2020-06', '2020-07', '2020-08']
['2020-02', '2020-03', '2020-04'] ['2020-06', '2020-07', '2020-08']
['2020-03', '2020-04', '2020-05'] ['2020-06', '2020-07', '2020-08']

Below are the time periods used for wave 2

['2020-01', '2020-02', '2020-03'] ['2020-11', '2020-12', '2021-01']
['2020-02', '2020-03', '2020-04'] ['2020-11', '2020-12', '2021-01']
['2020-03', '2020-04', '2020-05'] ['2020-11', '2020-12', '2021-01']
['2020-04', '2020-05', '2020-06'] ['2020-11', '2020-12', '2021-01']
['2020-05', '2020-06', '2020-07'] ['2020-11', '2020-12', '2021-01']
['2020-06', '2020-07', '2020-08'] ['2020-11', '2020-12', '2021-01']
['2020-07', '2020-08', '2020-09'] ['2020-11', '2020-12', '2021-01']
['2020-08', '2020-09', '2020-10'] ['2020-11', '2020-12', '2021-01']

Below are the time periods used for wave 3
['2020-01', '2020-02', '2020-03'] ['2021-03', '2021-04', '2021-05']
['2020-02', '2020-03', '2020-04'] ['2021-03', '2021-04', '2021-05']
['2020-03', '2020-04', '2020-05'] ['2021-03', '2021-04', '2021-05']
['2020-04', '2020-05', '2020-06'] ['2021-03', '2021-04', '2021-05']
['2020-05', '2020-06', '2020-07'] ['2021-03', '2021-04', '2021-05']
['2020-06', '2020-07', '2020-08'] ['2021-03', '2021-04', '2021-05']
['2020-07', '2020-08', '2020-09'] ['2021-03', '2021-04', '2021-05']
['2020-08', '2020-09', '2020-10'] ['2021-03', '2021-04', '2021-05']
['2020-09', '2020-10', '2020-11'] ['2021-03', '2021-04', '2021-05']
['2020-10', '2020-11', '2020-12'] ['2021-03', '2021-04', '2021-05']
['2020-11', '2020-12', '2021-01'] ['2021-03', '2021-04', '2021-05']
['2020-12', '2021-01', '2021-02'] ['2021-03', '2021-04', '2021-05']

Below are the time periods used for wave 4
['2020-01', '2020-02', '2020-03'] ['2021-07', '2021-08', '2021-09']
['2020-02', '2020-03', '2020-04'] ['2021-07', '2021-08', '2021-09']
['2020-03', '2020-04', '2020-05'] ['2021-07', '2021-08', '2021-09']
['2020-04', '2020-05', '2020-06'] ['2021-07', '2021-08', '2021-09']
['2020-05', '2020-06', '2020-07'] ['2021-07', '2021-08', '2021-09']
['2020-06', '2020-07', '2020-08'] ['2021-07', '2021-08', '2021-09']
['2020-07', '2020-08', '2020-09'] ['2021-07', '2021-08', '2021-09']
['2020-08', '2020-09', '2020-10'] ['2021-07', '2021-08', '2021-09']
['2020-09', '2020-10', '2020-11'] ['2021-07', '2021-08', '2021-09']
['2020-10', '2020-11', '2020-12'] ['2021-07', '2021-08', '2021-09']
['2020-11', '2020-12', '2021-01'] ['2021-07', '2021-08', '2021-09']
['2020-12', '2021-01', '2021-02'] ['2021-07', '2021-08', '2021-09']
['2021-01', '2021-02', '2021-03'] ['2021-07', '2021-08', '2021-09']
['2021-02', '2021-03', '2021-04'] ['2021-07', '2021-08', '2021-09']
['2021-03', '2021-04', '2021-05'] ['2021-07', '2021-08', '2021-09']
['2021-04', '2021-05', '2021-06'] ['2021-07', '2021-08', '2021-09']

Forecasting spreading mutations. The list of forecast mutations was generated by calculating Epi Score on the most recent three months of data and taking the top 200 ranked mutations. The threshold of 200 mutations was chosen based on the analysis presented in **fig. S5**.

Definition of variants of concern. Variants of concern were defined as those specified by the CDC, plus additional mutations which occurred at a rate of 80% of the most prevalent variant in the lineage*(3)*:

Alpha, B.1.1.7: H69-, V70-, Y144-, N501Y, A570D, D614G, P681H, T716I, S982A, D1118H

Beta, B.1.351: D80A, D215G, L242-, A243-, L244-, K417N, E484K, N501Y, D614G, A701V
Gamma, P.1: L18F, T20N, P26S, D138Y, R190S, K417T, E484K, N501Y, D614G, H655Y, T1027I, V1176F
Epsilon, B.1.427, B.1.429: S13I, W152C, L452R, D614G
Delta, B.1.617.2: T19R, T95I, G142D, E156del, E156G, F157del, R158G, R158del, L452R, T478K, D614G, P681R, D950N
+ AY lineages: V70F, Y145H, Q173H, A222V, W258L, K417N, A701S, T791I, A1078S, V1104L, D1153Y, D1259Y
Omicron: A67V, H69del, V70del, T95I, G142D, V143del, Y144del, Y145del, N211del, L212I, ins214EPE, G339D, S371L, S373P, S375F, K417N, N440K, G446S, S477N, T478K, E484A, Q493R, G496S, Q498R, N501Y, Y505H, T547K, D614G, H655Y, N679K, P681H, N764K, D796Y, N856K, Q954H, N969K and L981F.

For Omicron, due to the small sample size at the time of publication, it was necessary to account for un-sequenced sites in calculating mutation prevalence. This required re-running variant calling from read data, instead of using the summaries provided by GISAID. Mutations G446S, K417N, N440K, and N764K are included with such a correction.

SARS-CoV-2 pseudotyped VSV production and neutralization. To generate SARS-CoV-2 pseudotyped vesicular stomatitis virus, Lenti-X 293T cells (Takara) were seeded in 10-cm dishes for 80%. next day confluency. The next day, cells were transfected with a plasmid encoding for SARS-CoV-2 S-glycoprotein (YP_009724390.1) harboring a C-terminal 19 aa truncation and the D614G or D614G + S494P mutations using TransIT-Lenti (Mirus Bio) according to the manufacturer's instructions. One day post-transfection, cells were infected with VSV(G*ΔG-luciferase) (Kerafast) at an MOI of 3 infectious units/cell. Viral inoculum was washed off after one hour and cells were incubated for another day at 37°C. The cell supernatant containing SARS-CoV-2 pseudotyped VSV was collected at day 2 post-transfection, centrifuged at 1000 x g for 5 minutes to remove cellular debris, aliquoted, and frozen at -80°C.

For viral neutralization, Vero E6 cells were seeded into black-walled, clear-bottom 96-well plates at 20,000 cells/well and cultured overnight at 37°C. The next day, 9-point 5-fold serial dilutions of antibodies were prepared in media. SARS-CoV-2 pseudotyped VSV was diluted 1:20 in media and added 1:1 to each antibody dilution. Virus:antibody mixtures were incubated for 1 hour at 37°C. Media was removed from the cells and 50 µL of virus:antibody mixtures were added to the cells. One hour post-infection, 100 µL of media was added to all wells and incubated for 17-20 hours at 37°C. Media was removed and 50 µL of Bio-Glo reagent (Promega) was added to each well. The plate was shaken on a plate shaker at 300 RPM at room temperature for 15 minutes and RLUs were read on an EnSight plate reader (Perkin-Elmer).

Fig. S1. Testing of the working definition. (**A**) The working definition of spreading mutations captures the expansion of variants of concern during wave 3 at the country level. Leading variants of concern at the time are denoted by colored bars on the left-hand side. Green: Alpha B.1.1.7 ; Yellow: Gamma P.1; Blue: Beta B.1.351; Pink: Epsilon B.1.427/B.4.429. The emergence of E484K in association with Alpha B.1.1.7 is depicted in (A). Epsilon (B.1.427/B.4.429) mutations are also observed to spread across multiple countries. (**B**) (Next page) Previously unidentified (at the time) potential spreading mutations are presented separately. The x-axis countries are ordered left to right according to decreasing number of GISAID submissions being represented.

A

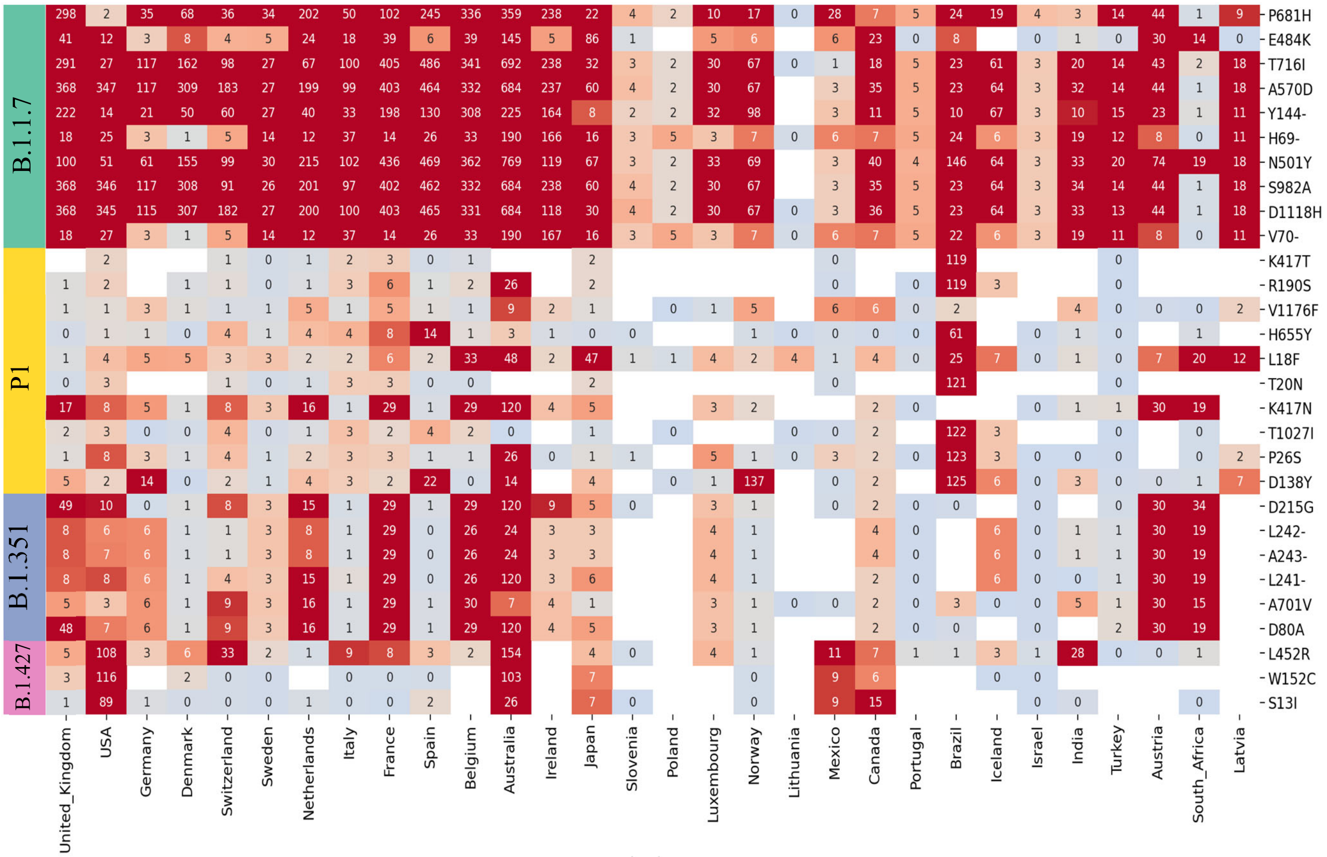

B

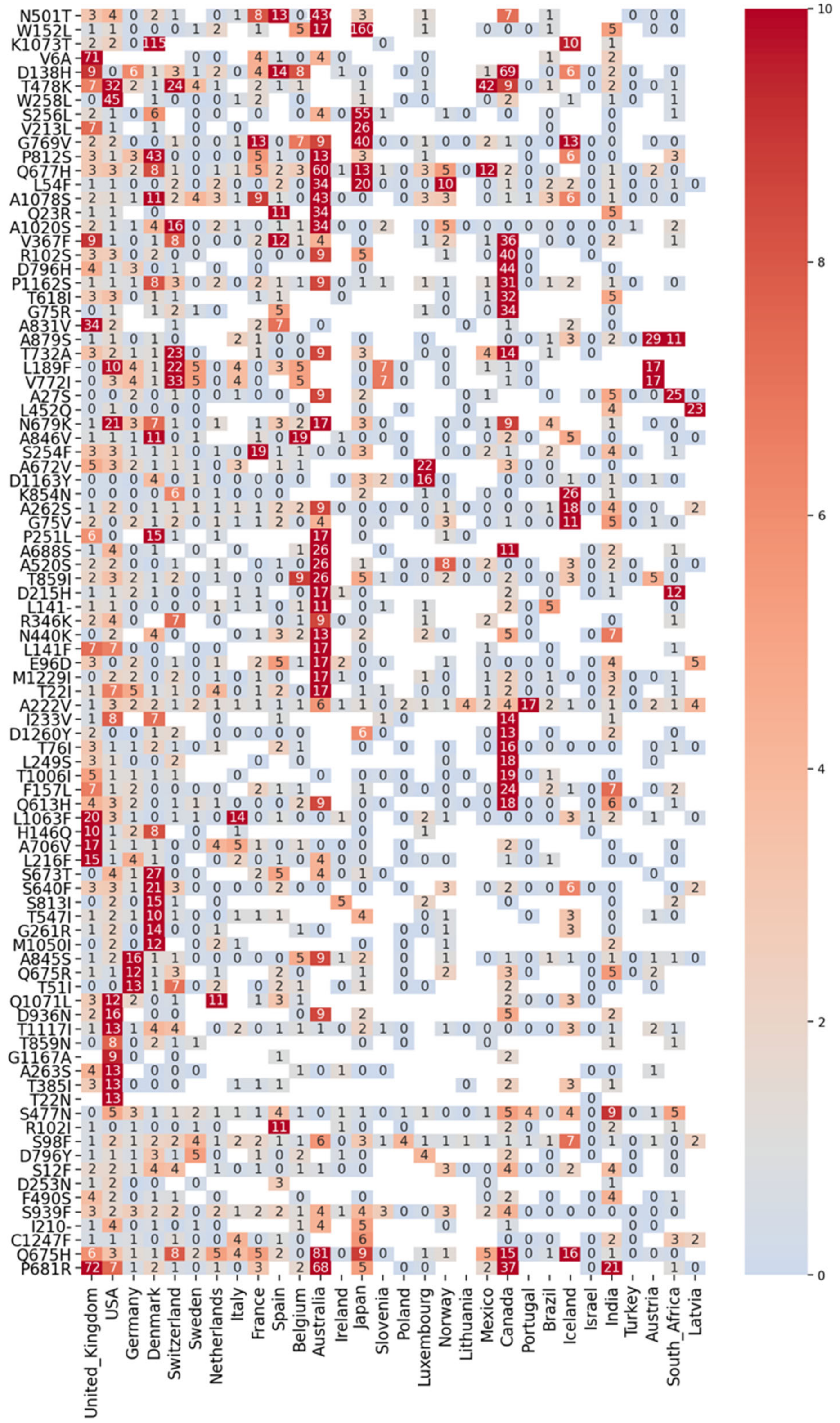

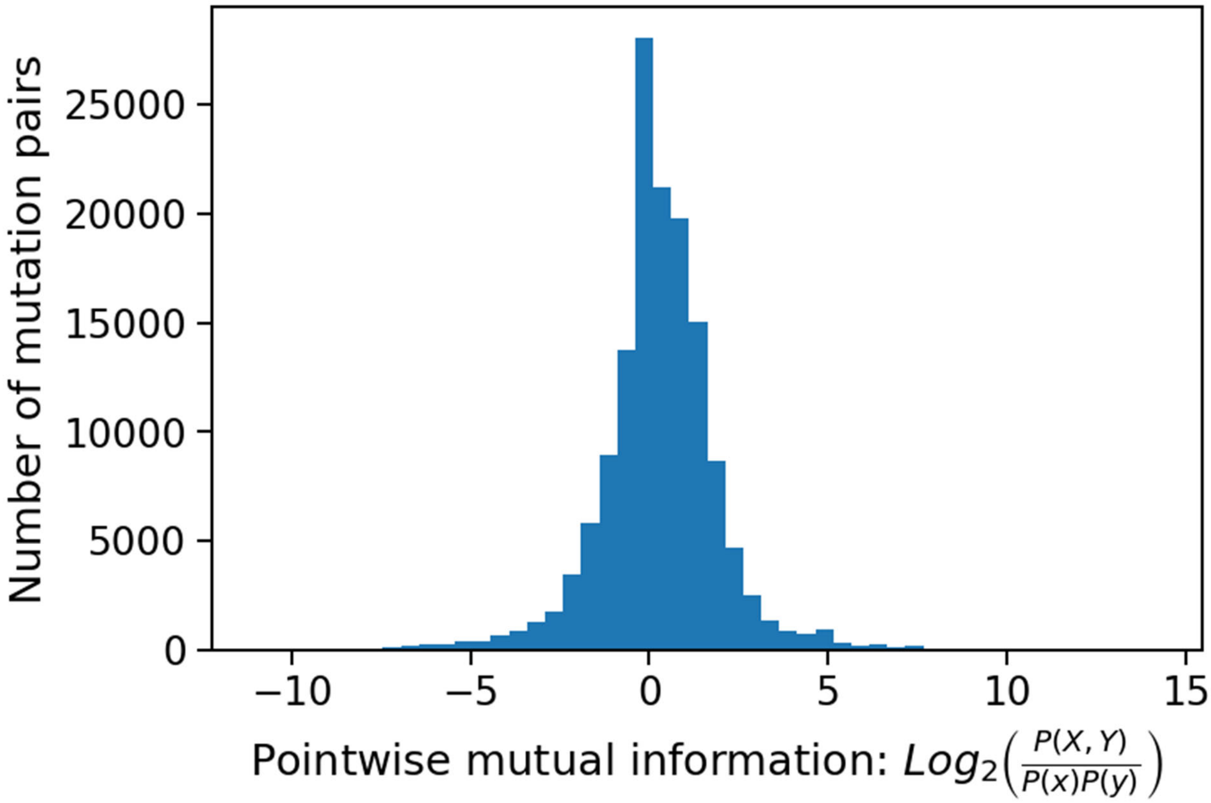

Fig S2. Epistasis between spreading mutations. We measured the $\log_2$ pointwise mutual information between all pairs of spreading mutations. Mutations at the same site or with an expected prevalence below 1/100,000 were excluded.

A

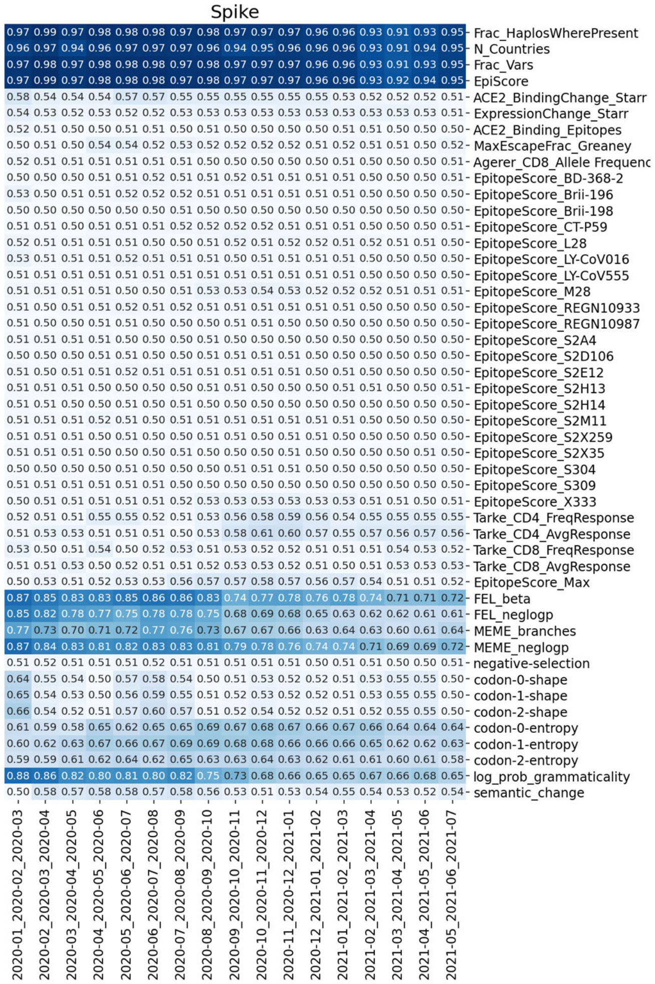

B

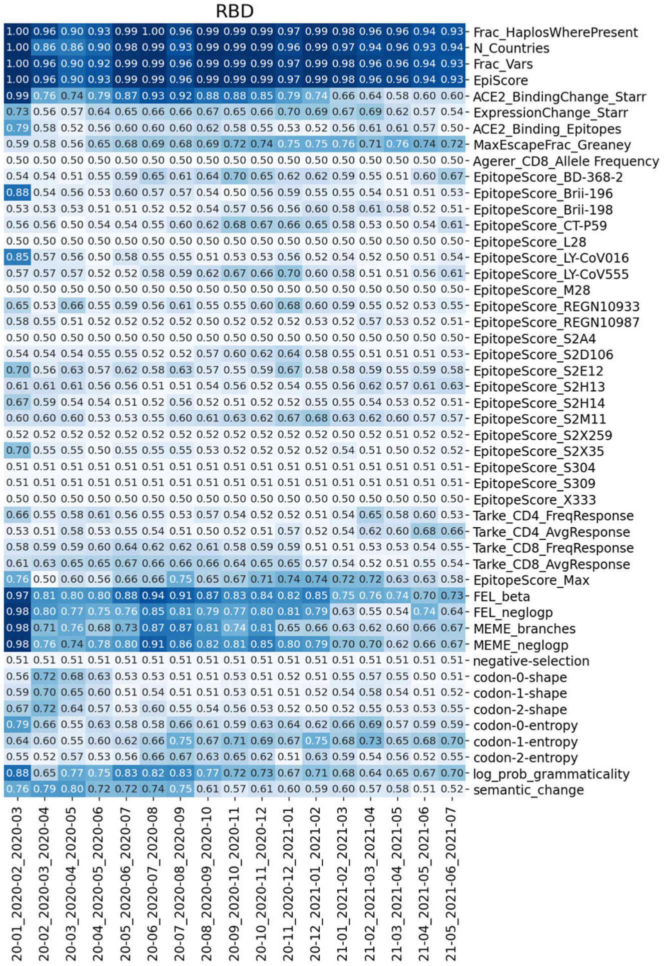

C

	MaxEscapeFrac_Greaney	EpitopeScore_Max
2020-01_2020-02_2020-03	3.3E-01	9.7E-02
2020-03_2020-04_2020-05	2.8E-01	1.6E-01
2020-05_2020-06_2020-07	1.9E-02	3.1E-02
2020-07_2020-08_2020-09	2.2E-02	2.3E-03
2020-09_2020-10_2020-11	9.6E-04	9.4E-03
2020-11_2020-12_2021-01	1.1E-04	2.6E-04
2021-01_2021-02_2021-03	6.2E-08	5.0E-06
2021-03_2021-04_2021-05	5.6E-06	1.2E-02
2021-05_2021-06_2021-07	8.0E-05	9.6E-02

Fig. S3. Changes in predictiveness over time. Trends in RBD (**A**) and Spike (**B**) AUROC for all variables, over 10 sliding window periods. Baseline analysis ROCs correspond to the 08/20-10/20 vs 11/20-01/21 analysis. (**C**) the p-values for difference from random prediction over time, for the immune escape variables. Explanations of variable names can be found in data file S1.

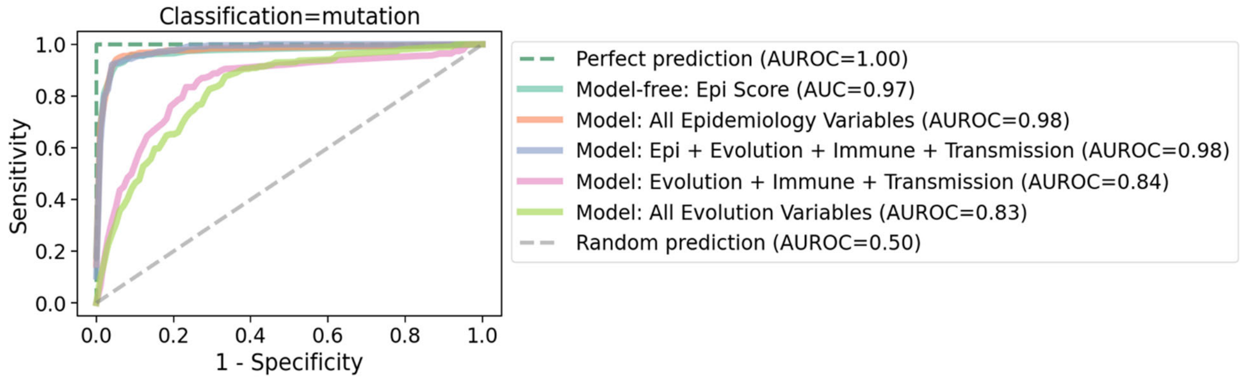

Fig. S4. Machine learning (ML) models on combined features do not improve Epi Score's AUROC. ROC curves for identifying which mutations will spread during the baseline analysis period (see Fig. 1A). "Model" analyses use a logistic regression model on feature sets, whereas "Model-free" approaches generate a single score without machine learning. Cross-validation was performed by stratifying by the clade on which each mutation predominantly appeared. To ensure comparability, these same cross-validation splits were used for the scoring step of the model-free approach. Specifically, ROC curves were generated for each test fold. The averaged curves and the areas under them are presented in this figure.

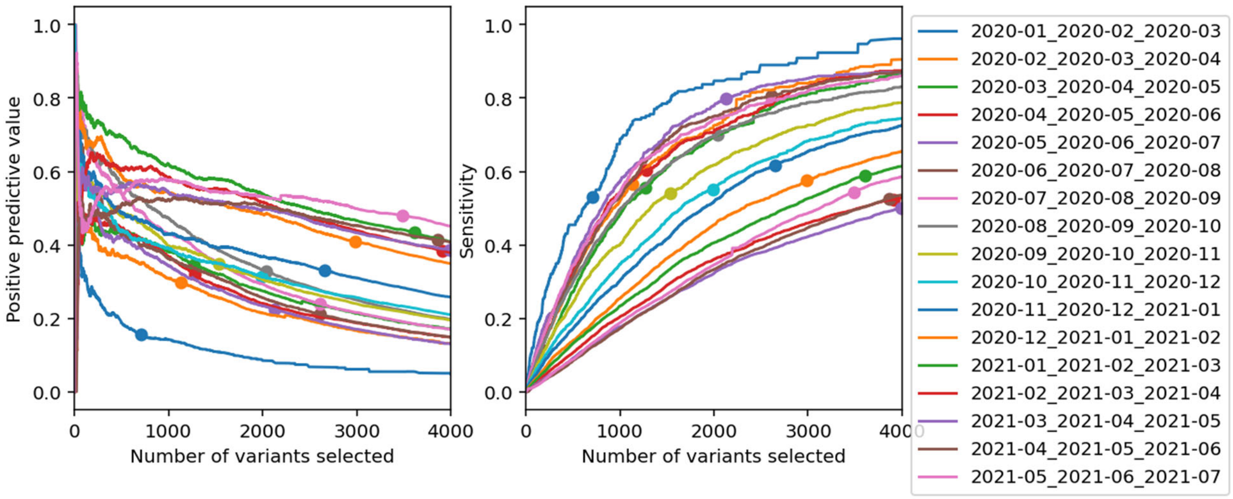

Fig. S5. Selecting the number of mutations to forecast as spreading. To understand the tradeoff between the fraction of *observed* spreading mutations correctly forecast (sensitivity), and the fraction of *predicted* spreading mutations that are correct (positive predictive value), we examined these quantities as a function of the number of top-scoring mutations forecasted to spread. We repeated this analysis across time windows, denoted by the three months prior to the cutoff. We found that taking the top 5% of mutations according to their Epi Score achieved reasonable sensitivity (~50%) and maintained a positive predictive value of between 20 and 60% across time windows.

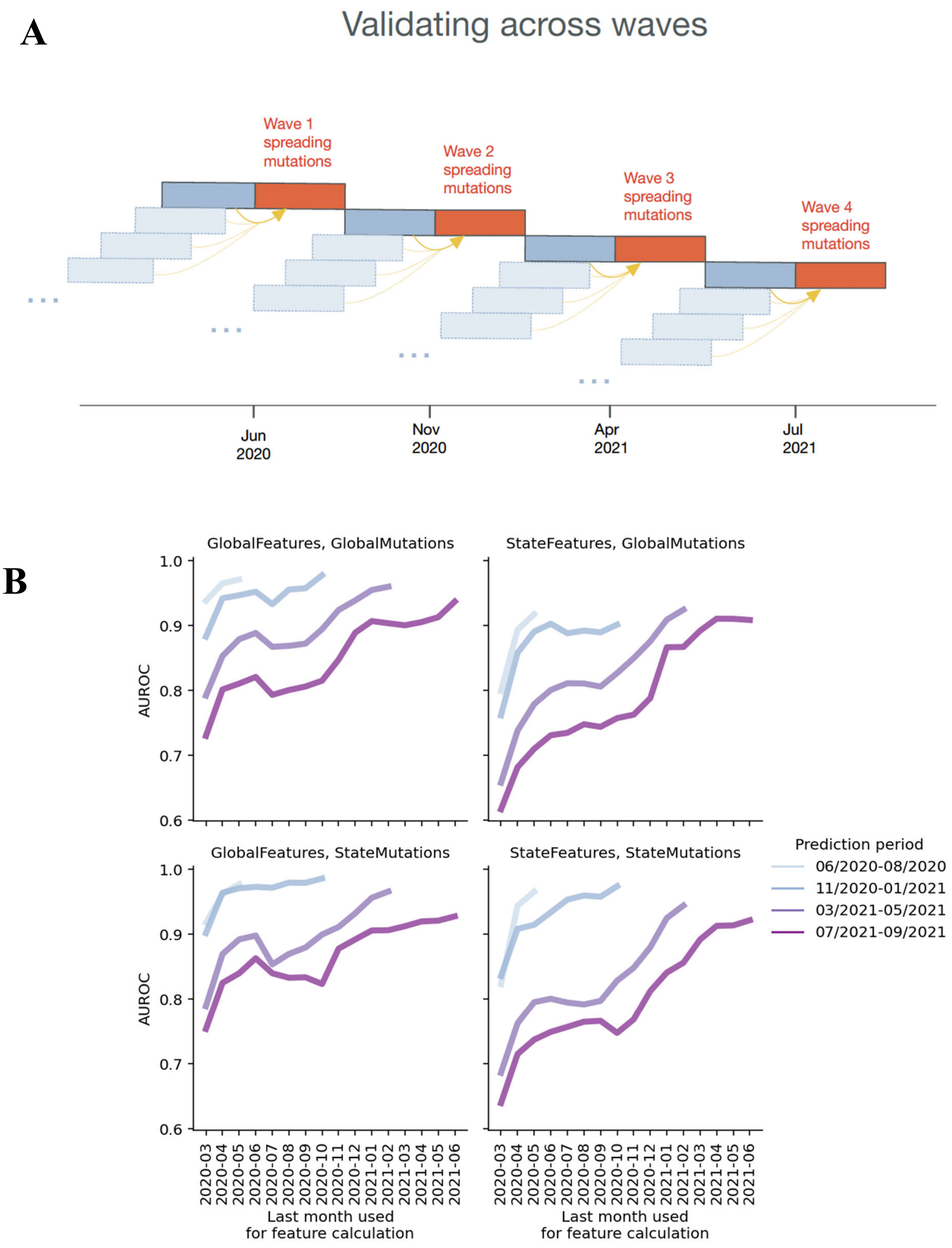

Fig. S6. Predicting local and global spreading mutations across pandemic waves. (**A**) An illustration of the analysis whereby, for each successive wave of the pandemic, spreading mutations are predicted using progressively earlier feature calculation windows. Both spreading mutations and calculated features can be derived from global data or data within the United States only. (**B**) Predictive performance (AUROC) for predicting mutations that are spreading globally ("GlobalMutations", top row) as well as within the United States ("StateMutations", bottom row), as predicted by features calculated globally (left column) and within the United States (right column), respectively. The analysis is repeated for four waves of the pandemic (hues; see legend) using data a varying number of months in advance (x-axis).

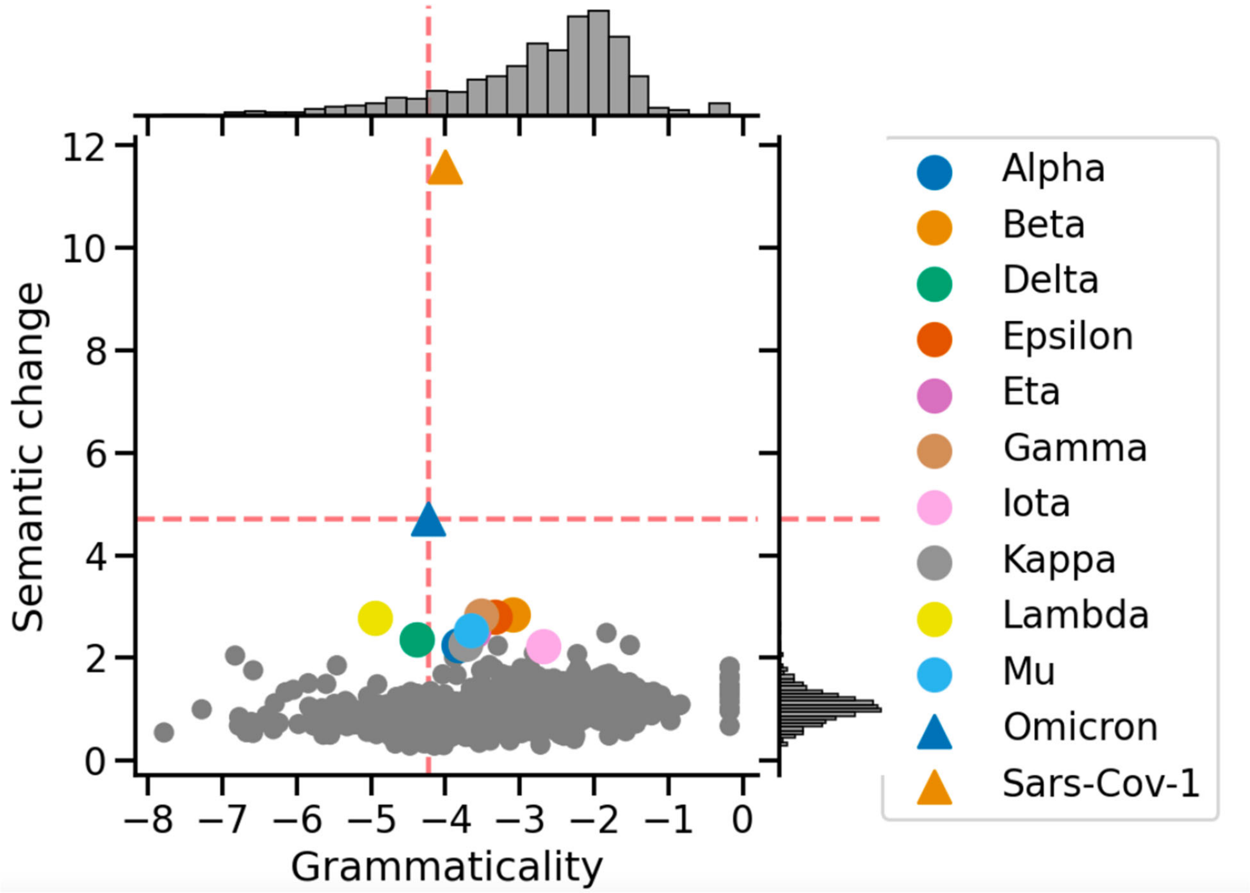

Fig. S7. Language model scores of VOCs/VBMs, Omicron, and SARS-CoV-2. Points in grey are a sample of sequences the model was trained on. Colored points show grammaticality and semantic change values for VOCs/VBMs, Omicron, and SARS-CoV-1. Dotted lines depict the values for Omicron. Histograms plot the marginal distributions of grammaticality and semantic change.

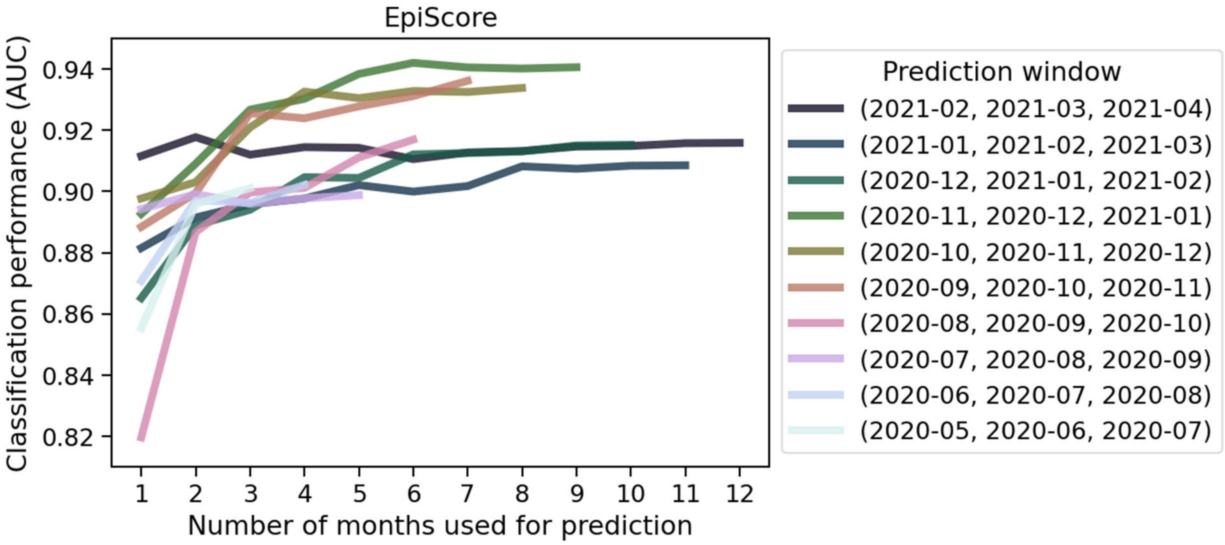

Fig. S8. The effect of the length of the feature calculation window on predictive performance. Colors indicate different time periods in which spreading mutations are predicted. The x-axis shows the number of months prior to this period used for feature calculation, and the y axis presents the classification performance (AUROC).

Table S1. The AUROCs for each variable complemented with the Epi Score. Performance (AUROC) for Epi Score + the listed variable is measured both within the RBD and across the whole spike protein. P-values represent difference from the best performing epidemiological (Epi Score) variable in bold. Variables above the bolded row indicate nominal complementarity within the RBD.

	**Spike AUC**	**Spike AUC *P* value**	**RBD AUC**	**RBD AUC *P* value**
**MEME_neglogp**	0.953	0.084	0.985	0.314
**FEL_neglogp**	0.954	0.55	0.984	0.51
**EpitopeScore_REGN10933**	0.954	0.195	0.984	0.513
**FEL_beta**	0.953	0.034	0.983	0.807
**Frac_Vars**	0.955	0.64	0.983	0.307
**N_Countries**	0.955	0.446	0.983	0.52
**EpitopeScore_S304**	0.955	0.349	0.983	1
**EpitopeScore_S2A4**	0.954	0	0.983	1
**EpitopeScore_L28**	0.956	0.42	0.983	1
**Agerer_CD8_Allele Frequency**	0.954	0.008	0.983	1
**EpitopeScore_X333**	0.956	0.149	0.983	1
**EpiScore**	0.955	1	0.983	1
**EpitopeScore_M28**	0.957	0.099	0.983	1
**EpitopeScore_S2X259**	0.955	0.081	0.983	1
**EpitopeScore_S309**	0.955	0.971	0.983	1
**MEME_branches**	0.953	0.075	0.983	0.96
**negative-selection**	0.955	0.464	0.983	0.48
**Frac_HaplosWherePresent**	0.955	0.11	0.983	0.48
**EpitopeScore_LY-CoV016**	0.955	0.668	0.983	0.916
**codon-2-shape**	0.956	0.329	0.982	0.72
**MaxEscapeFrac_Greaney**	0.954	0.145	0.982	0.244
**EpitopeScore_Brii-196**	0.954	0.148	0.982	0.746
**EpitopeScore_REGN10987**	0.955	0.927	0.982	0.425
**codon-1-shape**	0.956	0.277	0.982	0.619
**EpitopeScore_S2E12**	0.955	0.333	0.982	0.609
**log_prob_grammaticality**	0.951	0	0.982	0.557
**Tarke_CD4_FreqResponse**	0.954	0.797	0.982	0.538
**codon-0-shape**	0.956	0.308	0.981	0.425
**EpitopeScore_S2H13**	0.954	0.272	0.981	0.374
**EpitopeScore_S2X35**	0.955	0.197	0.981	0.314
**codon-1-entropy**	0.949	0	0.981	0.167
**codon-0-entropy**	0.948	0	0.981	0.234
**EpitopeScore_S2H14**	0.955	0.391	0.981	0.343
**EpitopeScore_Brii-198**	0.954	0.144	0.981	0.247
**Tarke_CD8_FreqResponse**	0.951	0.001	0.981	0.329
**EpitopeScore_Max**	0.949	0	0.98	0.118
**semantic_change**	0.953	0.06	0.98	0.406

**EpitopeScore_LY-CoV555**	0.954	0.13	0.98	0.216
**EpitopeScore_CT-P59**	0.954	0.047	0.98	0.249
**codon-2-entropy**	0.952	0.073	0.98	0.181
**Tarke_CD8_AvgResponse**	0.951	0	0.98	0.218
**EpitopeScore_S2D106**	0.954	0.136	0.98	0.137
**EpitopeScore_BD-368-2**	0.954	0.097	0.98	0.107
**Tarke_CD4_AvgResponse**	0.954	0.536	0.979	0.22
**EpitopeScore_S2M11**	0.954	0.103	0.979	0.076
**ACE2_Binding_Epitopes**	0.954	0.213	0.977	0.037
**ExpressionChange_Starr**	0.953	0.001	0.976	0.089
**ACE2_BindingChange_Starr**	0.953	0.004	0.971	0.013
